# Eligibility and Awareness Regarding Metabolic Surgery in Patients With Type 2 Diabetes Mellitus in the Real-World Clinical Setting; Estimate of Possible Diabetes Remission

**DOI:** 10.3389/fendo.2020.00383

**Published:** 2020-06-05

**Authors:** Chrysi Koliaki, Evangelia Tzeravini, Eleftheria Papachristoforou, Ioanna Severi, Elina El Deik, Melina Karaolia, Marina Noutsou, Anastasia Thanopoulou, Aikaterini Kountouri, Konstantinos Balampanis, Vaia Lambadiari, Nicholas Tentolouris, Alexander Kokkinos

**Affiliations:** ^1^First Department of Propaedeutic Internal Medicine and Diabetes Center, Medical School, National and Kapodistrian University of Athens, Laiko General Hospital, Athens, Greece; ^2^Diabetes Center, Second Department of Internal Medicine, Medical School, Hippokratio General Hospital, National and Kapodistrian University of Athens, Athens, Greece; ^3^Second Department of Internal Medicine and Research Institute, Medical School, National and Kapodistrian University of Athens, Attikon General Hospital, Athens, Greece

**Keywords:** bariatric surgery, metabolic surgery, eligibility criteria, type 2 diabetes mellitus, obesity, diabetes surgery summit

## Abstract

Despite high-quality evidence highlighting metabolic surgery as an effective treatment option for type 2 diabetes mellitus (T2DM), the number of patients receiving bariatric surgery (BS) remains low. Since the introduction of the Diabetes Surgery Summit II (DSS-II) eligibility criteria, data on eligibility rates for BS in T2DM cohorts remain scarce. The aims of the present study were to examine in a real-world clinical setting: (i) what is the percentage of T2DM patients visiting diabetes outpatient clinics who meet the DSS-II eligibility criteria, (ii) how many of these have been informed about the option of BS, and (iii) what are the characteristics associated with eligibility and awareness of BS. Demographic, anthropometric, clinical and socioeconomic data were obtained for all patients with T2DM who were consecutively examined in the outpatient clinics of three large-volume university hospitals (*n* = 1167). A medical registry form was completed to screen for BS eligibility. Patients were considered eligible if the recommendation by DSS-II criteria was either to “consider” or “recommend” BS. Eligible patients were further inquired whether they had ever been informed about the option of BS by their physicians. The advanced DiaRem score (ADRS) was applied to eligible patients to assess their probability of achieving postoperative T2DM remission. A significant percentage of T2DM patients who are routinely assessed in outpatient clinics meet the DSS-II eligibility criteria (15.3%). Eligible patients are younger and more obese, have a shorter T2DM duration, worse glycaemic control and better renal function, compared to non-eligible ones. Among eligible patients, only 39.3% have been medically informed about the option of BS. Informed patients are younger and more severely obese than non-informed ones. A significant percentage of non-informed patients (35%) have an ADRS ≤10, indicating a considerable probability for T2DM remission after BS, and are thus deprived of this opportunity due to lack of appropriate medical counseling. Screening and awareness of BS remain an unmet need in current T2DM management. Future research should focus on intensifying screening for BS eligibility at every medical visit and promoting evidence-based clinical recommendations for patients expected to benefit the most.

## Introduction

A large number of randomized clinical trials and high-quality prospective matched cohort studies over the past years have demonstrated the potential of bariatric or alternatively metabolic surgery to induce sustainable weight loss and provide substantial metabolic benefits in patients with obesity and type 2 diabetes mellitus (T2DM) (1–8). In the short and medium term, a significant amount of weight is lost, T2DM may completely regress, and cardiometabolic risk factors are dramatically improved. In the long term, bariatric surgery (BS) may achieve durable weight loss, prevent T2DM and cancer, mitigate life-threatening T2DM-related complications, improve overall glycaemic control minimizing the need for glucose-lowering medications, and reduce total and T2DM-related mortality (9, 10). Despite the wealth of evidence in the field of metabolic surgery and the magnitude of anticipated benefits, the number of patients receiving BS as T2DM treatment remains low. One possible reason for this low penetration of BS into diabetes care may be the failure of physicians to systematically screen for eligibility criteria and communicate efficiently the benefits of BS for appropriately selected patients (11).

Until recently, the most commonly applied eligibility criteria for BS were those established by the National Institutes of Health (NIH) in 1992 (12). Based on these criteria, eligible for BS are patients with morbid obesity, i.e., a body mass index (BMI) ≥40 kg/m^2^ regardless of their health status, and patients with a BMI between 35 and 40 with at least one severe obesity-related comorbidity such as T2DM, cardiovascular risk factors or non-alcoholic fatty liver disease (NAFLD). Patients with a BMI <35 or 35–40 without clinically relevant comorbidities are considered non-eligible according to NIH criteria. More than two decades later, the International Federation for the Surgery of Obesity and Metabolic Disorders (IFSO) shifted the focus of eligibility away from BMI, and recommended that BS should be further indicated for patients with a BMI >30 and comorbidities such as recent onset T2DM (13, 14). According to the IFSO, any indication for BS should consider metabolic comorbidities (T2DM), psychiatric symptoms, quality of life and functional limitations related to obesity. The terms bariatric or metabolic surgery should be thus replaced by the term “surgery for obesity and weight-related diseases” to capture the fact that these surgeries can improve and even cure obesity and weight-related conditions (13). In the same direction, in a position statement released in 2018, the American Society for Metabolic and Bariatric Surgery (ASMBS) revised its 2012 recommendations and urged the consideration of BS for individuals with a BMI between 30 and 35 (15). In June 2016, a number of leading international diabetes organizations focused on T2DM and issued new guidelines for the treatment of patients with obesity and T2DM, integrating BS into the proposed T2DM treatment algorithm. These guidelines were developed during the second Diabetes Surgery Summit (DSS-II), an international consensus conference, and were endorsed by numerous scientific societies all around the world (16). According to these guidelines, BS should be recommended to treat T2DM in patients with a BMI ≥40 kg/m^2^ (grade III obesity), regardless of their level of glycaemic control or complexity of glucose-lowering regimens, and also in patients with a BMI 35–39.9 kg/m^2^ (grade II obesity), if hyperglycemia cannot be controlled despite optimal lifestyle and medical treatment. BS should be further considered as an option to treat T2DM in patients with a BMI 30–34.9 kg/m^2^ (grade I obesity), if adequate glycaemic control cannot be achieved despite maximally intensified antidiabetic treatment, including injectable agents (16). Beyond BMI and glycaemic control criteria as determined by the DSS-II, eligibility for BS further requires the absence of serious life-threatening health conditions such as irreversible cardiopulmonary or other end-organ failure, metastatic or inoperable malignancy, active drug or alcohol abuse, and severe untreated psychiatric illness (17).

Since the introduction of the DSS-II criteria, data on eligibility rates for BS in T2DM cohorts are scarce. The aims of the present study were to examine in a real-world clinical setting: (i) what is the percentage of patients with T2DM visiting diabetes outpatient clinics of large university hospitals who meet the BS eligibility criteria proposed by DSS-II, (ii) how many of the eligible patients have been informed by their physicians about the option of BS, and (iii) what are the demographic, anthropometric and clinical characteristics associated with eligibility and awareness of BS among T2DM patients. We further assessed the possibility of T2DM remission in eligible patients using a recently proposed scoring system.

## Materials and Methods

### Study Population and Design

We performed an observational cross-sectional study in a large number of patients with T2DM who were consecutively examined in the diabetes outpatient clinics of three large-volume university hospitals within the time period 03/2019-12/2019 (*n* = 1167). The academic hospitals participating in this study were Laiko, Hippokrateio and Attikon General Hospital of Athens (Department of Internal Medicine and Diabetes Center), all three representing certified centers of excellence for diabetes research and clinical practice. Detailed demographic, anthropometric, clinical and socioeconomic data were obtained for all patients. These data included age, gender, BMI calculated as body weight in kg divided by height in meters squared (kg/m^2^), known duration of T2DM, current glycosylated hemoglobin (HbA1c) as a marker of glycaemic control, recent laboratory tests (within the last month) including serum creatinine to estimate glomerular filtration rate (GFR) based on the MDRD (Modification of Diet in Renal Disease) equation (18), current antidiabetic treatment with information on dose and frequency of glucose-lowering agents, T2DM-related comorbidities and complications, detailed drug history beyond antidiabetic treatment, presence of advanced heart failure defined as stages III and IV of the New York Heart Association (NYHA) staging system, presence of advanced renal disease using the GFR threshold of <30 ml/min, presence of advanced liver disease and alcohol abuse, history of malignancies and serious mental or psychiatric disorders requiring treatment.

All data were obtained by means of a structured registry form which was specifically designed to screen for BS eligibility. T2DM patients were considered eligible if the recommendation by DSS-II criteria was either to “consider” or “recommend” BS (based on BMI and glycaemic control). Optimal medical treatment for each patient was defined by experienced diabetologists in accordance with the updated evidence-based American Diabetes Association (ADA) clinical guidelines for T2DM pharmacologic treatment (19). Patients with age-related (>65 years old), health-related (advanced heart, liver, kidney disease, malignancy, uncontrolled mental/psychiatric disorder, eating disorders, alcohol abuse) or other socioeconomic reasons precluding surgery (lack of supporting environment, low level of education, severely impaired cognitive capacity) were considered to be non-eligible. The advanced Diabetes Remission score (Ad DiaRem), comprising pre-operative clinical variables such as age, HbA1c, insulin treatment, number of glucose-lowering agents and T2DM duration, was applied to all eligible patients to assess their probability of achieving T2DM remission after BS (20). For T2DM patients with an Ad DiaRem score of 0-5, 6-10, 11-15, 16-20, and >20, the probability of experiencing T2DM remission within the first 5 years after Roux-en-Y Gastric Bypass (RYGB) is estimated to be 100, 74.1, 28.6, 18.5, and 0%, respectively (21). The eligible patients were further inquired whether they had ever been informed about the option of BS as a treatment modality for their T2DM by their physicians in the diabetes outpatient clinics.

Before enrollment, all participants were thoroughly informed about the rationale and the aims of the study and gave written informed consent to the study protocol, which was approved by the Ethics Committees of all three participating hospitals, and conducted according to the World Medical Association's Declaration of Helsinki (amended in 2013). No patient refused to participate, so the drop-out rate was 0%.

### Statistical Analysis

Descriptive statistics were applied to present the demographic, anthropometric, and clinical characteristics of the study population. For continuous quantitative variables, data are presented as mean ± SD. For non-continuous quantitative variables such as T2DM duration and Ad DiaRem score, data are presented as median values plus interquartile range [25th-75th percentile]. For categorical variables, data are presented as absolute (n) and relative (%) frequencies. Comparisons between groups were performed with the non-parametric Mann-Whitney-Wilcoxon test for independent samples for continuous, and the Pearson's chi-square test for categorical variables. The level of statistical significance was set at 0.05, as appropriate. Statistical analysis was performed with the SPSS software package version 20.0 (Chicago, IL, USA).

## Results

Table 1 summarizes the major demographic, anthropometric and clinical characteristics of the study population. The mean age of the study participants was 65 ± 11 years. Nearly half of the studied patients (51.4%) had an advanced age, exceeding 65 years. Female patients represented 40.8% of the study population. More than half of T2DM patients in our cohort had obesity. Among obese patients, the majority had grade I obesity (BMI 30–34.9 kg/m^2^), almost a fourth had grade II obesity (BMI 35–39.9 kg/m^2^), and a fifth had morbid or grade III obesity (BMI ≥40 kg/m^2^). With respect to antidiabetic treatment, 57.5% of patients received only oral glucose-lowering agents, and 40.5% were treated with insulin (prandial, basal or mixture). The median duration of T2DM in our cohort was 11 years, while the mean HbA1c of the enrolled patients was 7.0 ± 1.4%, reflecting a relatively short T2DM duration and adequate glycaemic control. Regarding T2DM-related comorbidities, a significant number of patients reported hypertension and dyslipidemia, and a considerable number of patients reported a history of coronary heart disease (CHD). The prevalence of advanced heart failure (NYHA III/IV) in our cohort was 2.8%. The eligibility rate for BS according to DSS-II criteria was 15.3% in the total study population (*n* = 179 eligible patients), whereas the eligibility rate within the subgroup of T2DM patients with obesity was 29.1%.

**Table 1:** Demographic, anthropometric and clinical characteristics of the study population.

**Descriptive characteristics**	***N* = 1167 patients with T2DM**
Age (years)	65 ± 11
Female gender, *n* (%)	476 (40.8)
BMI (kg/m^2^)	31.3 ± 6.5
Obesity, *n* (%)	611 (52.4)
• Grade I, *n* (%)	345 (56.5)
• Grade II, *n* (%)	149 (24.4)
• Grade III, *n* (%)	117 (19.2)
Estimated GFR (ml/min)	79.7 ± 23.2
HbA1c (%)	7.0 ± 1.4
Known T2DM duration (years)	11 [5-18]
Antidiabetic treatment	
• Oral glucose-lowering medications, *n* (%)	671 (57.5)
• Treatment with insulin, *n* (%)	473 (40.5)
• Treatment with metformin, *n* (%)	990 (84.8)
• Treatment with sulfonylureas, *n* (%)	99 (8.5)
• Treatment with GLP-1RAs, *n* (%)	236 (20.2)
• Treatment with DPP-4 inhibitors, *n* (%)	417 (35.7)
• Treatment with SGLT2 inhibitors, *n* (%)	194 (16.6)
History of CHD, *n* (%)	265 (22.7)
Hypertension, *n* (%)	837 (71.7)
Dyslipidemia, *n* (%)	964 (82.6)
Heart failure NYHA III/IV, *n* (%)	33 (2.8)
**Eligibility for BS by DSS-II**, ***n*** **(%)**	**179 (15.3)**
**Eligibility for BS among obese patients**, ***n*** **(%)**	**179 (29.1)**

*For quantitative variables, data are presented as mean ± SD. For categorical variables, data are presented as absolute (n) and relative (%) frequencies. Data for known duration of T2DM are presented as median value plus interquartile range [25th-75th percentile]. Hypertension and dyslipidemia were defined based on the need for anti-hypertensive and hypolipidemic drug treatment, respectively. BMI, Body Mass Index; BS, bariatric surgery; CHD, coronary heart disease; DPP-4, dipeptidyl-peptidase 4; DSS-II, Diabetes Surgery Summit II; GFR, glomerular filtration rate; GLP-1RAs, glucagon-like peptide 1 receptor agonists; HbA1c, glycosylated hemoglobin 1c; NYHA, New York Heart Association; SGLT2, sodium-glucose cotransporter 2; T2DM, type 2 diabetes mellitus*.

### Comparison Between Eligible and Non-eligible Patients

Compared to non-eligible patients (NE), the eligible patients of our cohort (E) were more than a decade younger, significantly more obese by nearly 10 kg/m^2^, displayed worse glycaemic control as reflected by a higher HbA1c, better renal function, and had a shorter self-reported T2DM duration (8 vs. 12 years, *p* < 0.001). There was a marginally significant trend for a predominance of the female gender in eligible patients (*p* = 0.055). Furthermore, eligible patients were more frequently insulin-treated, possibly due to their poorer metabolic control (*p* = 0.03), and had a significantly lower prevalence of advanced heart failure, end-stage renal disease and mental or psychiatric disorders compared to NE patients (*p* < 0.05 for all). Among the eligible patients, 7.6% had an Ad DiaRem score 0-5, indicating a probability of 100% for achieving T2DM remission within the next 5 years following RYGB, 32.8% had a score 6-10, suggesting a probability of 74.1% for achieving T2DM remission, and 59.7% had a score >10, corresponding to a probability of only 23% for achieving T2DM remission after BS. Within the group of eligible patients, 39.3% declared that they had been previously informed by their physicians about the treatment option of BS.

Table 2 presents the comparison of major demographic and clinical characteristics between E and NE diabetic patients for BS according to the DSS-II eligibility criteria.

**Table 2:** Comparison of demographic and clinical characteristics between eligible (E) and non-eligible (NE) diabetic patients for metabolic surgery according to DSS-II eligibility criteria.

**Characteristics**	**Eligible patients (E) (*n* = 179)**	**Non-eligible patients (NE) (*n* = 988)**	***p*-value (E vs. NE)**
**Age (years)**	**54** **±** **8**	**67** **±** **10**	**<0.001**
Female sex (%)	47.5	39.8	0.055
**BMI (kg/m**^**2**^**)**	**39.5** **±** **6.3**	**29.8** **±** **5.3**	**<0.001**
**Estimated GFR (ml/min)**	**87** **±** **24**	**78** **±** **23**	**<0.001**
**HbA1c (%)**	**7.7** **±** **1.8**	**6.9** **±** **1.2**	**<0.001**
**T2DM duration (years)**	**8 [2-15]**	**12 [5-18]**	**<0.001**
**Insulin treatment (%)**	**48**	**39.3**	**0.03**
History of CHD (%)	20.6	23.1	0.5
Hypertension (%)	70.5	72.1	0.8
Dyslipidemia (%)	77.8	83.7	0.056
**Advanced heart failure (%)**	**0**	**3.2**	**0.016**
**Advanced kidney disease (%)**	**0**	**2.7**	**0.029**
**Psychotic disorder under treatment (%)**	**0**	**6.4**	**0.001**
Alcohol abuse (%)	1.1	1.3	0.9
**For eligible patients only**			
Informed about the option of BS (%)	39.3	NA	
Advanced DiaRem score	12 [9-18]	NA	
• Advanced DiaRem score 0-5 (%)	7.6	NA	
• Advanced DiaRem score 6-10 (%)	32.8	NA	
• Advanced DiaRem score >10 (%)	59.6	NA	

*For continuous quantitative variables, data are presented as mean ± SD. For categorical variables, data are presented as relative frequencies (%). For non-continuous quantitative variables such as T2DM duration and advanced DiaRem score, data are presented as median values plus interquartile range [25th-75th percentile]. Comparisons between groups were performed with non-parametric Mann-Whitney test for independent samples. Statistically significant differences are denoted with bold font*.

*Advanced DiaRem score 0-5 indicates a probability of 100% for achieving T2DM remission within the next 5 years following RYGB*.

*Advanced DiaRem score 6-10 indicates a probability of 74.1% for achieving T2DM remission within the next 5 years after RYGB*.

*Advanced DiaRem score >10 indicates a probability of only 23% for achieving T2DM remission within the next 5 years after RYGB*.

*NA, not applicable; BMI, Body Mass Index; BS, bariatric surgery; CHD, coronary heart disease; Diarem score, diabetes remission score; DSS-II, Diabetes Surgery Summit II; GFR, glomerular filtration rate; HbA1c, glycosylated hemoglobin 1c; RYGB, Roux-en-Y gastric bypass; T2DM, type 2 diabetes mellitus*.

### Comparison Between Informed and Non-informed Patients

**Figure 1:**
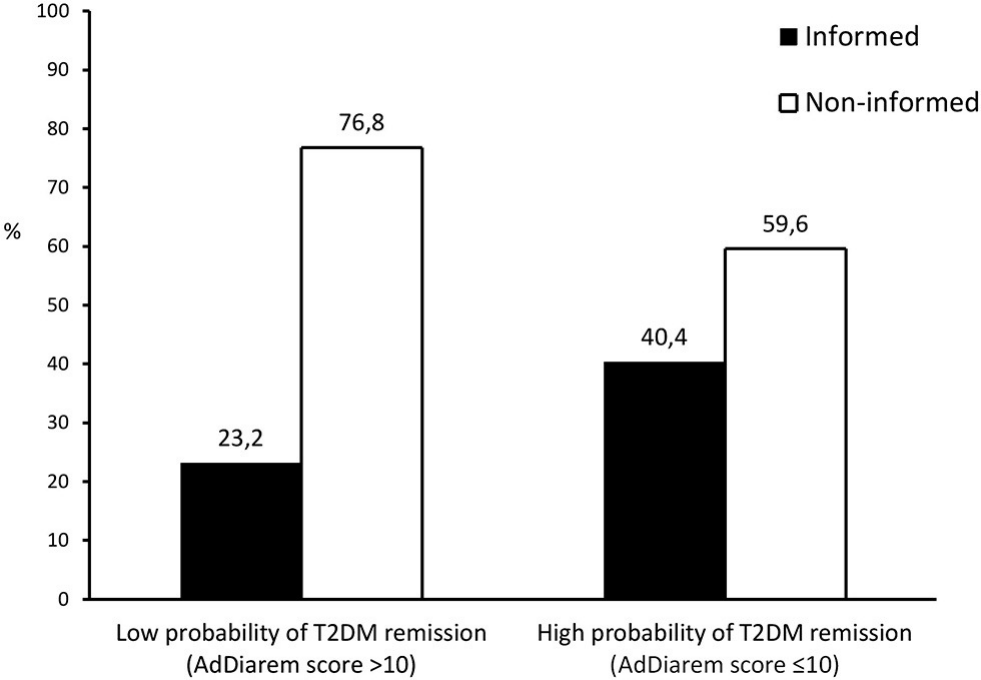
Graphical presentation of the percentage of patients having been informed about the treatment option of metabolic surgery among those with high vs. low probability of T2DM remission within the first 5 years following bariatric surgery, stratified according to the advanced DiaRem[[Inline Image]] score. AdDiarem score, advanced diabetes remission score; T2DM, type 2 diabetes mellitus.

Eligible patients who had been informed about the option of BS (I) were significantly younger (*p* < 0.001), more obese (*p* < 0.001) and reported a shorter T2DM duration (*p* = 0.009), compared to eligible patients who had never been medically informed about BS (NI), as summarized in Table 3. Glycaemic control and renal function did not differ significantly between I and NI groups. Informed patients had a significantly lower Ad DiaRem score (10 vs. 14, *p* = 0.016) compared to non-informed patients, indicating a higher probability to achieve T2DM remission after BS. Accordingly, the percentage of patients having an Ad DiaRem score ≤10 and thus a probability of achieving post-operative T2DM remission of up to 74%, was significantly higher among informed vs. non-informed patients (54.3 vs. 34.6%, *p* = 0.047). Among the eligible patients with high probability of achieving T2DM remission after BS (Ad DiaRem score ≤ 10), the percentage of informed patients was 40.4 vs. 23.2% among those unlikely to experience postoperative T2DM remission, as shown in Figure 1.

**Table 3:** Comparison of characteristics between informed (I) and non-informed (NI) eligible diabetic patients for metabolic surgery.

**Characteristics**	**Informed patients (I) (*n* = 68)**	**Non-informed patients (NI) (*n* = 111)**	***p*-value (I vs. NI)**
**Age (years)**	**51** **±** **10**	**56** **±** **7**	**<0.001**
Female sex (%)	44.1	49.5	0.49
**BMI (kg/m**^**2**^**)**	**43.1** **±** **7.3**	**37.1** **±** **4.3**	**<0.001**
Estimated GFR (ml/min)	91 ± 26	85 ± 23	0.16
HbA1c (%)	7.5 ± 1.7	7.9 ± 1.9	0.17
**T2DM duration (years)**	**4 [1-14]**	**9 [5-16]**	**0.009**
**Advanced DiaRem score**	**10 [7-15]**	**14 [10-18]**	**0.016**
**Advanced DiaRem score** **≤10 (%)**	**54.3**	**34.6**	**0.047**

*For continuous quantitative variables, data are presented as mean ± SD. For categorical variables, data are presented as relative frequencies (%). For non-continuous quantitative variables such as T2DM duration and Advanced DiaRem score, data are presented as median values plus interquartile range [25th-75th percentile]. Comparisons between groups were performed with non-parametric Mann-Whitney test for independent samples. Statistically significant differences are denoted with bold font*.

*Advanced DiaRem score ≤10 indicates a probability of up to 74.1% for achieving T2DM remission within the next 5 years following bariatric surgery*.

*BMI, Body Mass Index; DiaRem score, diabetes remission score; GFR, glomerular filtration rate; HbA1c, glycosylated hemoglobin 1c; T2DM, type 2 diabetes mellitus*.

## Discussion

Summarizing the major findings of the present study, a significant number of patients with T2DM who are routinely assessed in diabetes outpatient clinics of large university hospitals meet the DSS-II eligibility criteria for BS (15.3%). Eligible patients are younger and have a higher degree of obesity, report a shorter T2DM duration and display worse glycaemic control and better renal function, compared to non-eligible patients. Among eligible patients, only around 40% have been informed by their physicians about the option of BS. Informed patients are younger and have more severe obesity than non-informed patients. A significant percentage of unaware patients (35%) have an advanced DiaRem score ≤10, indicating a considerable probability of 74% to achieve T2DM remission within the next 5 years after BS, and are thus deprived of this opportunity due to lack of appropriate medical counseling. These data highlight the fact that both screening and awareness of BS remain an unmet need in the current clinical setting of T2DM management.

We report a considerable percentage of patients with T2DM who are eligible for BS. This relates to the DSS-II eligibility criteria that we applied, which consider not only the extent of obesity but also the adequacy of glycaemic control to determine eligibility for BS in patients with T2DM. As opposed to the previously applied NIH criteria which were more BMI-centric and precluded the option of BS for patients with T2DM and mild obesity (BMI <35) (12), the DSS-II criteria expand BS eligibility to a broader spectrum of patients with obesity and T2DM who remain suboptimally controlled despite intensified medical and lifestyle treatment (16). Several studies have emphasized the need to extend BS eligibility beyond stringent BMI thresholds and instead focus on clinically relevant outcomes to maximize benefits for patients. Strict BMI cut-offs have been consistently found inappropriate for BS prioritization, considering that even non-eligible patients based on BMI may experience comparable benefits in terms of T2DM prevention, improvement of cardiovascular risk factors and mortality reduction with eligible patients (22). To date, the majority of studies addressing the epidemiology of BS eligibility have used either NIH or other evidence-based criteria sets (22, 23). Studies applying the DSS-II eligibility criteria in real-world T2DM populations are lacking, and our study aims to contribute to this literature gap.

Our study delineated the profile of eligible T2DM patients for BS by comparing demographic, anthropometric and clinical characteristics between eligible and non-eligible groups. Compared to non-eligible patients, eligible ones were found to be younger, more obese, with shorter T2DM duration, better renal function and poorer glycaemic control. The higher BMI and HbA1c found in eligible patients should be expected, considering that the degree of obesity and glycaemic control are both components of the definition of the DSS-II criteria. The younger age of eligible patients could be also expected, since advanced age (>65 years old) precludes eligibility, and a large number of T2DM patients in our cohort (nearly 50%) were considered non-eligible due to their advanced age. This also probably points to clinical inertia on the part of the medical profession, namely that doctors do not recommend BS to eligible patients for many years, until finally these patients become ineligible due to their age. The shorter reported T2DM duration in eligible patients could be partly related to their younger age. Although the duration of T2DM is not officially part of the definition of eligibility criteria for BS, it should be always considered, since patients with long-standing T2DM have a lower probability to achieve T2DM remission after BS (10). The better renal function of eligible patients could be explained by their younger age, shorter T2DM duration and fewer comorbidities.

An important finding of our study was that less than 40% of eligible T2DM patients responded positively to the question whether they had ever been informed by their physicians about the option of BS. This means that ~60% of T2DM patients who fulfill the eligibility criteria for BS and would theoretically benefit from BS in terms of T2DM treatment, overall health and quality of life improvement, are not made aware of this option due to lack of appropriate medical consultation and recommendation. Informed patients were found to be significantly younger and more obese than non-informed patients, suggesting that physicians tend to consider BS primarily for younger patients with extreme levels of adiposity. As a result of this misconception, eligible patients who are older or have less severe obesity are not promptly informed about BS, an omission which is very unfortunate for them, because as time goes by, their age will advance further, comorbidities may accumulate, T2DM duration will increase and glycaemic control may possibly deteriorate, all of which might eventually render these patients either non-eligible for BS or poor candidates for post-operative T2DM remission. An additional important observation of our study was that among eligible T2DM patients who had never been informed about the option of BS (unaware), around 35% displayed a favorable Ad DiaRem score (≤10), indicating a considerable likelihood of achieving T2DM remission within the next 5 years after BS. The significantly higher percentage of patients with a favorable prospect of T2DM regression after BS in informed vs. non-informed patients (54.3 vs. 34.6%) is definitely a positive statement, but still, a significant number of patients who are highly likely to experience T2DM remission in the next years after BS, miss this opportunity and remain diabetic and poorly controlled due to lack of awareness. Possible reasons for this huge gap in awareness are mainly related to the inadequate education of physicians about BS, ignorance of current eligibility criteria, low familiarity with BS modalities and their risks and benefits, lack of interest or confidence in BS, lack of time and reluctance to undertake the postoperative monitoring of bariatric patients (24–26), as demonstrated in a Greek study in doctors of various medical specialties, which assessed their attitudes and perceptions about BS and reported an alarmingly limited penetration of BS in the medical community (27). These data emphasize the urgent need to promote education of health care professionals in the field of obesity management and make them more knowledgeable, confident and familiar with weight management options such as BS. It would be meaningful to pursue this goal by implementing changes in the curriculum of medical schools to teach medical students how to interact with patients with obesity and optimally manage their obesity-related health conditions, as shown by innovative pilot studies in undergraduate medical students (28).

Our data reveal an unmet need in T2DM clinical management, and complement previously published data addressing several unmet needs in both obesity and T2DM management (29). Despite the indisputable weight loss efficacy of BS, only a small fraction of qualifying patients with obesity undergo BS, partly due to the associated perioperative risks, reimbursement issues, and prevalent misconceptions. Nationwide datasets suggest a huge gap between the number of patients with obesity who are eligible for BS and those who ultimately receive it, and they reveal differences in demographic parameters between eligible and operated patients, raising concerns about possible inequalities in access to BS in certain population subgroups (30). In support of these findings, an Irish study has shown that the current provision of bariatric surgical services meets much less than 0.1% of the current need based on eligibility rates in older community-dwelling adults (23). In this context, it should be emphasized that BS should be performed by experienced surgeons in specialized centers providing multidisciplinary teams and long-term follow up. Therefore, BS eligibility should be “adapted” to the capacity and availability of such units.

This study has certain limitations. Its cross-sectional design, the descriptive methodology, the subjectivity of determining individual eligibility for BS with respect to defining optimal medical treatment (based however on evidence-based clinical guidelines), and the lack of a nationally representative study sample, are the major of these. On the other hand, our study has important strengths. It adds to the limited literature applying the novel DSS-II eligibility criteria to prioritize BS in inadequately controlled diabetic patients, reflects everyday clinical practice by studying real-world patients with T2DM and varying levels of obesity assessed routinely in diabetes outpatient clinics, and conveys the simple but important message that awareness of BS remains low despite high eligibility, even among patients who are expected to benefit the most.

Taken together, the present study concludes that a significant proportion of patients with T2DM who are routinely assessed in diabetes outpatient clinics are appropriate candidates for BS, but only a minority of them have been informed by their physicians about this option. Physicians tend to recommend BS only in young patients with extreme levels of adiposity. These data highlight the fact that screening and awareness of BS in the clinical setting of T2DM management remain an unmet need possibly due to insufficient education and clinical inertia. Future research should focus on intensifying screening for BS eligibility criteria at every medical visit and promoting accurate and evidence-based clinical recommendations for patients expected to benefit the most.

## Data Availability Statement

The raw data supporting the conclusions of this article will be made available by the authors, without undue reservation.

## Ethics Statement

The studies involving human participants were reviewed and approved by Ethics Committees of Laiko General Hospital, Athens Greece, Hippokratio General Hospital, Athens, Greece, and Attikon General Hospital, Athens, Greece. The patients/participants provided their written informed consent to participate in this study.

## Author Contributions

CK analyzed data and prepared the manuscript. ET collected, tabulated and analyzed data and reviewed the manuscript. EP, IS, EE, MK, MN, AT, AKou, KB, and VL collected and tabulated data and reviewed the manuscript. NT analyzed data and reviewed the manuscript. AKok designed and coordinated the study, analyzed data and reviewed the manuscript. All authors approved the final version of the submitted manuscript.

## Conflict of Interest

The authors declare that the research was conducted in the absence of any commercial or financial relationships that could be construed as a potential conflict of interest.
